# Editorial – Hypoxia and Reoxygenation: From Basic Science to Bedside

**DOI:** 10.3389/fped.2015.00086

**Published:** 2015-10-19

**Authors:** Michele Samaja, Giuseppina Milano

**Affiliations:** ^1^Department of Health Science, University of Milan, Milan, Italy; ^2^Centre Hospitalier Universitaire Vaudois, Lausanne, Switzerland

**Keywords:** intermittent hypoxia, hypoxia-inducible factor, hypoxic preconditioning, reoxygenation, OSAS, myocardial ischemia

A condition with inadequate oxygen supply to the tissues, hypoxia plays a pivotal role in the pathology of cyanotic congenital heart defects and several adult diseases as myocardial infarction, stroke, cancer, diabetes, aging, and pulmonary obstruction. Most cell responses to hypoxia are modulated by hypoxia-inducible factors [HIFs (1)], DNA-binding transcription factors that mediate hypoxia adaptation through activation of a multitude of genes encoding proteins needed to improve tissue oxygen delivery, energy metabolism, efficient management of hypoxia-induced stress and regulation of apoptosis, autophagy, and cell cycle. The reoxygenation that follows hypoxia usually induces bursts of reactive oxygen species, which not only cause the oxidative damage central in the pathophysiology of hypoxia/reoxygenation (HReox) injury but also activate signaling mechanisms that in part synergize and in part oppose those induced by hypoxia. Consequently, it becomes often difficult to distinguish what is attributable to hypoxia and what to the reoxygenation that follows hypoxia. A new research frontier may foster clues to understand the mechanisms underlying HReox injury and to identify appropriate targets to design interventions aimed at reducing the toll of this injury in several diseases.

One example is paradigmatic. For many years, hypoxia was believed to be associated with cardioprotection. However, it is now evident that it is not hypoxia, but HReox, the factor providing cardioprotection (2), or hypoxic preconditioning (3). In hearts exposed to hypoxia, alterations in several signaling paths (4, 5) converge into deleterious phenotypes as right ventricular hypertrophy and impaired ability to resist ischemia/reperfusion (6). These findings are corroborated by the clinical observation that the outcome of surgery for repair of cyanotic congenital heart defects is complicated by myocardial damage due to HReox at the moment of the institution of cardiopulmonary bypass with elevated oxygen content, followed by ischemia/reperfusion injury when heart is arrested to perform the intra-cardiac repair (7). HReox is related to the intermittent hypoxia (IH) paradigm, e.g., repeated exposure to short periods of hypoxia 5–10 times/day. Although displaying protective features in the favor of musculoskeletal, pulmonary, central nervous, and cardiopulmonary systems (8), IH may have Janus-like features (9): on the one hand, IH is a form of hypoxic preconditioning (10); on the other hand, it may become dangerous and pave the road to obstructive sleep apnea syndrome (OSAS) (11, 12), which causes harmful redox stress (13).

The purpose of this “Research Topic,” attracting articles dealing with causes and effects of HReox, aims at better understanding the underlying pathophysiology and mechanism in the cardiopulmonary system to improve the outcome of clinical management in several classes of pediatric and adult patients.

Verges et al. (14) reviewed if and how hypoxic preconditioning is a valid therapeutic modality in healthy subjects (hematology, ventilation, cardiovascular system, and metabolic status) and in patients affected by cardiovascular (acute myocardial infarction, chronic coronary artery disease, heart failure), neurological (stroke, spinal cord injury), respiratory (OSAS, chronic obstructive pulmonary diseases), and metabolic diseases. It appears that hypoxic preconditioning, or IH training, is distinguishable from dangerous OSAS-like IH essentially for intensity and frequency of hypoxia challenges. Indeed, the progression from protective to dangerous IH is a continuum based on intensity and physical characteristic of the HReox spikes. A role for CO_2_ handling is envisaged because, whereas protective IH is characterized by hypocapnic hypoxemia, OSAS-like IH is characterized by hypercapnic hypoxemia. In both cases, the molecular determinants for the cell response to HReox are primarily HIFs followed by HIF downstream responses involving GATA-4, K^+^ channels, various MAPK, EPO, and the PI3K/Akt pathways.

Serebrovskaya and Xi (15) started from the concept that IH might occur in early infancy in both preterm and term infants, and 2–4% of all children are affected by OSAS. There are numerous reports indicating adverse effects of IH on development, behavior, achievement, and cognition in OSAS children, although the exact causative relationship remains uncertain. On the other hand, well-controlled and moderate IH can be used in sick children for treating various forms of bronchial asthma, allergic dermatoses, autoimmune thyroiditis, cerebral palsy, and obesity. The review into the critical role of IH in childhood and comparison of the genesis of harmful vs. beneficial effects of IH leads to emphasize the dual aspect of IH leading to hypoxia preconditioning and OSAS-like IH. Not only CO_2_ handling but also hypoxia-induced polycythemia are identified as most important determinants for the divergent effects of these two paradigms.

Hashimoto and Shibasaki (16) clarified the role of HIFs in regulating the transcription of genes that mediate the response to hypoxia, especially in carcinogenesis and in the pathology of ischemic diseases. Unexpectedly, clinical trials targeted at the control of therapeutic angiogenesis after the administration of a single growth factor have yielded unsatisfactory or controversial results for a range of causes accurately examined in their review. However, the manipulation of HIF-2α, which plays an essential role in vascular remodeling, appears more promising for the treatment of ischemic diseases caused by arterial obstruction, where insufficient development of collateral vessels impedes effective therapy. Indeed, the eukaryotic initiation factor 3 subunit e (eIF3e)/INT6 has been identified as the molecular target that stabilizes specifically HIF-2α even under normoxia, thereby inducing the expression of several angiogenic factors, and reducing injury in ischemic limbs or cold-injured brains in animal models.

Farah and Reboul (17) focused into a most prominent factor in IH-downstream pathway, e.g., nitric oxide (NO). They describe the characteristics of the various NO synthase isoforms in providing NO, and the ways by which NO exerts its effects against I/R injury, through activation of the guanylate cyclase and protein kinase G pathway, or through S-nitrosylation of certain proteins. This view of the phenomenon related to the protective vs. harmful effects of NO opens a more rational understanding of the contribution and importance of NO with respect to the subcellular localization (especially with reference to sarcoplasmic reticulum and mitochondrial proteins), concentration (NO bioavailability may be considered advantageous below a threshold level but harmful above it), and timing (thus opening an interesting window for oral-based nitrite therapies to prevent I/R injury).

Wellmann et al. (18) described how both hypoxia and hyperoxia trigger focal necrosis in brain white matter giving raise to diffuse white matter diseases, and consequently disturb myelination in newborn infants during a critical developmental window prior to the onset of myelination. This may represent the underlying cause for an array of cognitive and behavioral diseases. Using various targeted experimental models, it has been shown that the molecular basis of such phenotypes, e.g., HIF-1α and HIF-2α, can be activated by both hypoxia (through the canonical β-catenin-Wnt signaling) and paradoxically by a situation that resembles hyperoxia (transgenic embryonic mice unable to express HIF-1α and HIF-2α). Such findings emphasize the importance of avoiding extremes of oxygen tension in the brains of preterm infants and in infants with congenital heart disease undergoing surgery.

In summary, these reviews converge into the view that appropriate management of HReox may help curing many diseases and may provide pediatric surgeons valuable tools to reduce hypoxia-induced complicacies (2, 19).

## Conflict of Interest Statement

The authors declare that the research was conducted in the absence of any commercial or financial relationships that could be construed as a potential conflict of interest.
